# Modified three-level techniques of retroperitoneal laparoscopic procedures to treat adrenal lesions for patients with BMI ≥ 25 Kg/m^2^

**DOI:** 10.7150/ijms.49574

**Published:** 2020-10-22

**Authors:** Minxiong Hu, Zesong Yang, Yuandong Chen, Guangbing Chen, Zhensheng Chen, Tao Li, Qingguo Zhu, Yongbao Wei, Liefu Ye

**Affiliations:** 1 Shengli Clinical Medical College of Fujian Medical University and Department of Urology, Fujian Provincial Hospital, Fuzhou 350001, China.; 2 Department of Urology, Fujian Provincial Hospital South Branch, Fuzhou 350028, China.; 3 Fujian Medical University, Fuzhou 350004, China.; 4 Department of Urology, Min Dong Hospital of Ningde City Ningde, Fujian Province, China.; 5 Department of Urology, Fuding Hospital Affiliated to Fujian University of Traditional Chinese Medicine, Fuding City, Fujian Province, China.

**Keywords:** adrenal gland, retroperitoneal laparoscopy, BMI, fat

## Abstract

**Objective:** To evaluate the modified Zhang's 'three-level' technique of retroperitoneal laparoscopic adrenalectomy (RLA) to treat adrenal lesions for patients with BMI of 25-30 Kg/m^2^.

**Methods:** A retrospective analysis was performed in all patients with BMI of 25-30 Kg/m^2^ in our hospital from January 2014 to December 2019. Those who underwent laparoscopic adrenal surgery were divided into two groups on the basis of the technique used: the Zhang's technique (the ZT group) and the modified technique (the MT group).

**Results:** Herein, 170 operations were included (ZT, 91 patients; MT, 79 patients). RLA was successfully performed in all of them. Compared with the ZT group patients, the MT group patients showed shorter operation time (*p* = 0.007), lesser intraoperative blood loss (*p* = 0.023), shorter operation time, earlier postoperative diet recovery (*p* < 0.001), shorter postoperative drainage time (*p* < 0.001) and shorter postoperative hospitalization period (*p* = 0.001). It was also worth noting that the unplanned total adrenalectomy rate was significantly less in the MT group than in the ZT group (0% vs. 10.8%, *p* = 0.020). There was no significant difference in the complications between the two groups (3.3% vs. 2.5%, *p* = 0.567).

**Conclusions:** We found that MT was a beneficial retroperitoneal laparoscopic treatment for adrenal lesions in patients who had a BMI of 25-30 Kg/m^2^. It may provide a reference for the treatment of adrenal surgical diseases in such patients.

## Introduction

Over the past three decades, laparoscopic adrenalectomy has replaced open adrenalectomy and become the gold standard for surgical treatment of most adrenal diseases 1. Transperitoneal laparoscopy is one of the most widely used adrenal approaches 2 because it allows for the best comprehensive observation of the adrenal region and its surrounding tissues. Gagner was the first to perform retroperitoneal laparoscopic adrenalectomy (RLA) in 1992 3. Both retroperitoneal and transabdominal laparoscopic treatments of benign adrenal tumours are now considered safe and effective, with similar outcomes 4. Even RLA has lesser postoperative pain and shorter hospitalization periods than the transabdominal approach 5. The retroperitoneal approach has its unique advantages, making it a valuable alternative method to treat the adrenal gland lesions 6. RLA has recently become more popular 7 and its advantage is that it allows direct access to the retroperitoneal cavity despite the small working space. Therefore, it avoids the abdominal cavity and limits the risk of vascular and other visceral injuries 8, thus resulting in less postoperative pain and shorter hospitalizations 5. In 2007, Zhang et al. reported their 'three-level' method of RLA, which involved programmed and simplified procedures of RLA 9. The learning curve of this method seems shorter 10, and it has become a widely accepted approach to treat adrenal surgical diseases in mainland China in the last decade.

During our clinical practice, we found the 'three-level' RLA suitable for most patients. However, it seems not an effective enough method to treat overweight and obese patients as their relatively thick peri-renal fat is makes the retroperitoneal space relatively narrow. Some patients have peri-renal fat adhesion, which makes it more difficult when separating the upper pole of the kidney from the peri-renal fat. Even after an effort-intensive process of separation, the adrenal glands are still invisible because of the upper pole fat. Thus, we apply this method of changing the third level approach to make the procedure more convenient to perform for overweight and obese patients.

## Materials and Methods

### Patients

We retrospectively included patients who had undergone adrenal surgery in our department from January 2014 to December 2019. Patients who met the following criteria were included: (a) RLA was performed for the patients with adrenal tumours or lesions; (b) patients with BMI ≥25 Kg/m^2^; (c) the surgical procedure was Zhang's standard three-level method or the modified one; (d) patients underwent CT or MRI scan of the abdomen before operation; (e) the patients accepted RLA by the same skilled surgeon in our department.

The exclusion criteria were that patients did not meet the above conditions at the same time, the patients were not willing to participate in this study or the data available was insufficient for analysis.

We analysed the effectives and peri-operative safety of our modified surgical approach compared with Zhang' s standard 'three-level' method for overweight and obese patients with BMI ≥25 Kg/m^2^ 11.

The study was approved from by the Ethics Committee of our hospital. Written informed consents were obtained from the guardians of these patients.

### Surgical approach

#### Key points of Zhang's standard 'three-level' technique (ZT)

**Figure 1:**
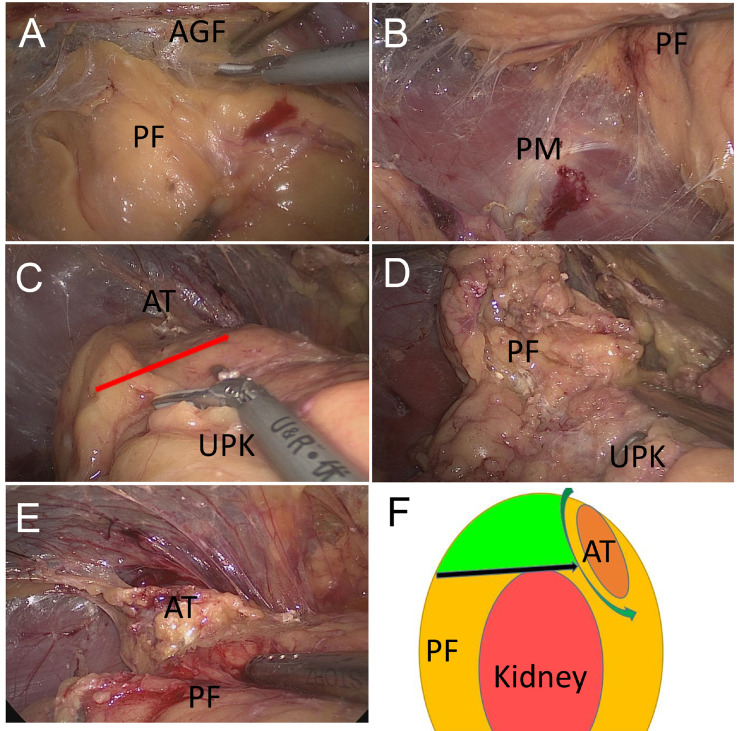
** Surgical approach of 'three-level' and modified 'three-level' methods.** (**A**) The first level: between anterior Gerota's fascia (AGF) and perinephric fat (PF) that located at the superomedial side of the kidney. (**B**) The second level: between lateral perinephric fat (PF) and psoas muscle (PM). (**C**) The third level (shown by the arrow): between the bottom of adrenal tumour (AT) and upper pole of the kidney (UPK). (**D**) When adherent or large perinephric fat (PF) exists, the third level is not easy to obtain and the adrenal tumour is not easy to access. (**E**) The modified third level: pull the perinephric fat (PF) away, and the adrenal tumour (AT) is visible clearly. (**F**) A diagram of the modified 'three-level' approach. The green area is the redundant perinephric fat (PF) in this technique. This fat can be used as a pull position to give enough operation view to discover the adrenal tumour (AT).

Preoperative preparations were made according to the patient's preoperative diagnoses. This technique was performed as per the protocol of Zhang et al. 9. Briefly, first, the retroperitoneal space was prepared. Then, three trocars were placed as working channels. The pressure of CO_2_ pneumoperitoneum was maintained at 11 mmHg (1 mmHg = 0.133 kPa). The extra-peritoneal fat was removed and Gerota's fascia was separated. Then, we performed the first separation of the relative avascular space between the adrenal fat capsule and the anterior Gerota's fascia (Fig.1A). Then, in the second separation level, the relatively avascular space was between the dorsal side of the adrenal fat capsule and the psoas muscle (Fig.1B). The third separation level was located between the surface of the upper adrenal capsule and the peri-renal fat, dissociating it with the lower adrenal pole (Fig. 1C).

#### Key points of modified three-level Technique (MT)

During our clinical practice, we have identified that when adherent or large peri-nephric fat exists, the traditional third level intervention is not easy to perform and the adrenal tumour is not easy to access (Fig. 1D). Thus, we modified the third level of this approach. The operation method of our modified third level was essentially the same as Zhang's standard three-level method, except that the separation was improved at the third separation level: the peripheral renal fat was dissected from the dorsal side of the upper pole to the adrenal gland. After reaching the adrenal gland, the adrenal gland and the adenoma were exposed along the edge of the adrenal gland (Fig.1E). The auxiliary hand could then pull the peri-renal fat outwards to create a large enough operation space at the upper pole of the kidney (Fig. 1F).

### Statistical analysis

Statistical software SPSS 24.0 was used for analysis. The independent sample t test or chi-square test was used for comparison. A *p* value of <0.05 was considered statistical significance.

## Results

### General demographic data

A total of 170 cases were included in this study. All patients successfully underwent endoscopic surgery, and none was converted to open surgery. There were 91 cases in the ZT group and 79 cases in the MT group. The ZT group had 45 men and 46 women, with an average BMI of 27.68 Kg/m^2^ ± 2.94 Kg/m^2^ and an average tumour diameter of 4.21 cm ± 1.29 cm. The MT group had 44 men and 35 women, with an average BMI of 27.45 Kg/m^2^ ± 1.89 Kg/m^2^ and an average tumour diameter of 4.27 cm ± 1.35 cm. In the MT group, the lesions were located on the left in 44 cases and on the right in 35 cases. There was no statistically significant difference between the two groups in terms of gender, BMI, tumour diameter, tumour type and left- and right-side comparison (all *p* > 0.05). Patients in the ZT group were marginally older than those in the MT group [ZT, 55.10 ± 10.89 years vs. MT, 51.52 ± 11.93 years *p* < 0.05] (Table 1).

### Comparison of peri-operative period between the two groups

The ZT group planned reserving 74 cases adrenal glands (82.2%) and 17 cases (17.8%) of planned adrenalectomy, but finally, there were nine cases (12.2%) accepted as unplanned adrenalectomy. The MT group planned reserving 61 cases of adrenal glands (77.2%) and 18 cases of planned total adrenalectomy (22.8%); no unplanned cases (0%) resulted in total adrenalectomy. Obviously, the proportion unplanned adrenalectomy in the MT group was lower (*p* < 0.05) (Table 2). The average operation time of the ZT group was 115.89 ± 34.94 min and blood loss were 45.22 ± 66.07 ml; the average postoperative recovery time was 1.63 ± 0.95 days. The drainage tube extraction time in the ZT group was 4.79 ± 2.21 days; its average postoperative hospitalisation period was 6.78 ± 3.74 days. The average operation time in the MT group was 102.49 ± 28.20 min and blood loss were 28.42 ± 20.06 ml; postoperative recovery time was 1.15 ± 0.48 days. The postoperative drainage tube extraction time was 3.47 ± 1.52 days, and the postoperative hospitalisation period was 4.78 ± 3.73 days. On comparing the two groups, it was found that compared with the ZT group, the MT group had a shorter operation time, lesser blood loss, shorter postoperative diet recovery time, earlier postoperative drainage tube removal and shorter postoperative hospitalisation period (all *p* < 0.05). The incidences of complications in both groups were very low (*p* > 0.05) (Table 3), including three cases (3.3%) in the ZT group (two cases of subcutaneous emphysema and one case of incisional hernia) and two cases (2.5%) in the MT group (one case of subcutaneous emphysema and one case of chylic leakage).

## Discussion

Over the past few decades, the proportion of overweight and obese individuals has been rising globally; this health concern is already widespread in high-income countries and shows an increasing trend in low- and middle-income countries as well. The growth rate of this concern is the highest in men and boys in Western high-income countries and in women and girls in Central Asia, the Middle East and North Africa 12. Being overweight or obese is a predictor of peri-renal fat adhesion, and a higher BMI indicates that peri-renal fat adhesion is more severe 13, 14. BMI was positively correlated with high operation time, more severe postoperative complications and prolonged hospitalisations 15. Fatty adhesions around the kidney increase the 'third level' 9 separation, which may damage the renal capsule, cause bleeding and poorly expose the surgical field, thus directly leading to prolonged operation time and even unplanned removal of the adrenal gland. The 'three-level' method was the basis of posterior laparoscopic adrenal surgery in China. A large number of clinical practices have found that the thickness of peri-renal fat in the general population is significantly different from that in overweight and obese people. We found that this 'three-level' method has limitations. The present study found that complete removal of the adrenal glands was not planned in 10.8% of overweight and obese patients; further, it was not planned in any patient in the MT group. This result suggests that the conventional 'three-level' method may be suitable for most patients, but it seems difficult to perform the 'three-level' method in overweight and obese patients, particularly in those with peri-renal fat adhesions. Therefore, for overweight and obese individuals, a modified surgical method may be necessary.

Several methods of posterior laparoscopic adrenal surgery currently exist, each with its advantages and disadvantages. Hu et al. searched for the central adrenal vein under the guidance of the renal pedicle or inferior vena cava by changing the position of the trocar and separated the relatively avascular space between the dorsal side of the peripheral renal fat capsule and Gerota's fascia 16. Moreover, some researchers proposed 'single layer' posterior laparoscopy to treat benign adrenal diseases 17. Compared with the 'three-level' method, the above two surgical methods do not dissociate the whole kidney, thus resulting in a lower degree of mobility, and the problem of the narrow retroperitoneal space remains as they cannot provide a wider surgical space. Although their operation may be successful, it may require more trocars and more extensive surgical experience. If complications such as a vascular injury occur during the operation, it may be more difficult to handle and would require greater expertise. Of course, more studies are needed to evaluate whether the modified 'three-level' technology is superior to these technologies in terms of the learning curve and technical advantages.

In our study, the age was different between these two groups (*p* < 0.05); the mean age of the ZT group individuals higher. This may be because of our small sample size. However, studies found that age was not an independent factor associated with prolonged time of surgery. Erbil et al. performed linear regression analysis using the operation time as the dependent variable, and they concluded that age was not an independent risk factor of the operation time 15. This was consistent with our clinical experience. Although the operation time seems to be obviously related to tumour diameter, tumour location and BMI among other factors, in our study, no statistically significant differences were observed between the two groups in these parameters, and thus, we believed that our data of the two groups were still comparable, and the results of this study may still be practically significant.

The limitation of this study was that it was a retrospective study with a small sample size. In addition, some of these patients may have to undergo an unplanned transfer to the modified technique because of the peri-renal fat adhesion. However, we did not make stratified analyses for patients with different kinds of fatty adhesions as well as the adrenal lesions located at the medial branch because of the limited cases included. Furthermore, we had no enough reason to explain the MT group patients had the earlier postoperative diet recovery compared the patients accepted the Zhang's techniques, even which may be related to shorter operation time and/ or less blood lose. We will conduct further research on these concerns after we accumulate more cases, and we hope more studies especially randomised ones to further valuate this improved technique in future.

## Conclusion

To our knowledge, this is the first report on the application of the modified 'three-level' technique in the laparoscopic adrenal gland surgery for overweight and obese patients. This technique may provide a reference for the treatment of adrenal surgical diseases in these patients.

## Figures and Tables

**Table 1:** General demographic data

	ZT	MT	*P* value
**Gender (n)**			
Male	45	44	0.374
Female	46	35	
Age (years)	55.10 ± 10.89	51.52 ± 11.93	0.042
BMI (Kg/m^2^)	27.68 ± 2.94	27.45 ± 1.89	0.548
**Tumour location (n)**			
Left	54	44	0.683
Right	37	35	
Tumour size (cm)	4.21 ± 1.29	4.27 ± 1.35	0.733
Tumour type (n)			
Incidental tumour	52	42	
Pheochromocytoma	2	5	1.000
Others	37	32	

ZT, Zhang's technique; MT, modified technique.

**Table 2:** The rates of unplanned adrenalectomy between the two groups

	Planned adrenalectomy	Planed adrenal sparing	Unplanned adrenalectomy	The rate of unplanned adrenalectomy (%)
ZT	17	74	9	10.8%
MT	18	61	0	0
*P* value		0.020		

ZT, Zhang's technique; MT, modified technique.

**Table 3:** Comparison of perioperative period between the two groups

	ZT	MT	*P* value
Operation time (min)	115.89±34.94	102.49 ± 28.20	0.007
Blood loss (ml)	45.22 ± 66.07	28.42 ± 20.06	0.023
Diet recovery (days)	1.63 ± 0.95	1.15 ± 0.48	<0.001
Drainage (days)	4.79 ± 2.21	3.47 ± 1.52	<0.001
Postoperative hospitalisations (days)	6.78 ± 3.74	4.78 ± 3.73	0.001
Complications (n)	3/91(3.3%)	2/79(2.5%)	0.567

ZT, Zhang's technique; MT, modified technique.
